# Real-World Registry on the Pharmacotherapy of Multiple Myeloma and Associated Renal and Pulmonary Impairments in the Greater Gulf Region: Protocol for a Retrospective Real-World Data Study

**DOI:** 10.2196/49861

**Published:** 2024-04-24

**Authors:** Abdulnaser Nourallah, Abdulrahman Alshehri, Ayman Alhejazi, Binyam Usman, Ghada ElGohary, Hafiz Malhan, Ibraheem Motabi, Khalil Al Farsi, Mohammed Alshuaibi, Mustaqeem Siddiqui, Rasha Ghonema, Ruba Yasin Taha, Tarek Abouzeid, Wesam Ahmed, Mohanad Diab, Ahmad Alhuraiji, Magdy Rabea, Mohamed Zahir Chouikrat

**Affiliations:** 1 Hematology/Medical Oncology Department, Almana General Hospital Alkhobar Saudi Arabia; 2 Hematology / Oncology Department, Aseer Central Hospital Abha Saudi Arabia; 3 Division of Adult Hematology and Hematopoietic Stem Cell Transplantation, Department of Oncology, King Abdulaziz Medical City Riyadh Saudi Arabia; 4 Department of Oncology, King Faisal Specialist Hospital and Research Center Jeddah Saudi Arabia; 5 Department of Medicine, Division of Oncology/Hematology, College of Medicine, King Saud University Medical City Riyadh Saudi Arabia; 6 Department of Adult Hematology, Prince Mohammed bin Nasser Hospital Jazan Saudi Arabia; 7 Adult Hematology & Bone Marrow Transplant Department, Comprehensive Cancer Center at King Fahad Medical City Riyadh Saudi Arabia; 8 Alfaisal University Riyadh Saudi Arabia; 9 Department of Hematology, Sultan Qaboos University Hospital Muscat Oman; 10 Adult Hematology and Oncology Divisions, Department of Medicine, King Abdul-Aziz Hospital Alahsa Saudi Arabia; 11 Hematology & Oncology Division at Sheikh Shakhbout Medical City (SSMC) Abu Dhabi Saudi Arabia; 12 Department of Hematology, Kuwait Cancer Control Center Kuwait Kuwait; 13 Department of Hematology-Bone Marrow Transplantation, National Centre for Cancer Care and Research (NCCCR), Hamad Medical Corporation (HMC) Doha Qatar; 14 Internal Medicine Department, Almouwasat Hospital Dammam Saudi Arabia; 15 Cleveland Clinic Abu Dhabi, Oncology Institute Abu Dhabi United Arab Emirates; 16 Cleveland Clinic Florida, Oncology Institute Florida, FL United States; 17 Hemato-oncology Department, Burjeel Hospital Abu Dhabi United Arab Emirates; 18 Medical Affairs Department, Sanofi Jeddah Saudi Arabia; 19 Medical Affairs Department, Sanofi Dubai United Arab Emirates

**Keywords:** Greater Gulf region, multiple myeloma, pulmonary dysfunction, renal impairment, RRMM, Real-world data

## Abstract

**Background:**

Multiple myeloma (MM) is the second-most common cancer among hematological malignancies. Patients with active disease may experience several comorbidities, including renal insufficiency and asthma, which may lead to treatment failure. The treatment of relapsed or refractory MM (RRMM) has been associated with multiple factors, causing a decline in progression-free survival as well as overall survival with subsequent lines of therapy. Data about the characteristics of this group of patients in the Greater Gulf region are lacking.

**Objective:**

The primary objective of this study is to describe the disease characteristics and various treatment approaches or regimens used in the management of patients with RRMM in the Greater Gulf region.

**Methods:**

We will conduct a regional, retrospective study collecting real-world and epidemiological data on patients with MM in countries of the Greater Gulf region. Medical records will be used to obtain the required data. Around 150 to 170 patients’ records are planned to be retrospectively reviewed over 6 months without any cross-sectional or prospective intervention. Cases will be collected from Saudi Arabia, the United Arab Emirates, Kuwait, Oman, and Qatar. Descriptive as well as analytical statistics will be performed on the extracted data. The calculated sample size will allow us to estimate the percentages of RRMM cases with acceptable precision while complying with the challenges in light of data scarcity. We will obtain a comprehensive description of the demographic profile of patients with MM; treatment outcomes; the proportion of patients with MM with renal impairment and asthma, chronic obstructive pulmonary disease, or both at the time of diagnosis and any subsequent point; and data related to treatment lines, regimens, and MM-associated morbidities.

**Results:**

Patient medical records were reviewed between June 2022 and January 2023 for eligibility and data extraction. A total of 148 patients were eligible for study inclusion, of whom 64.2% (n=95) were male and 35.8% (n=53) were female. The study is currently in its final stages of data analysis. The final manuscript is expected to be published in 2024.

**Conclusions:**

Although MM is a predominant hematological disease, data on its prevalence and patients’ characteristics in the Greater Gulf region are scarce. Therefore, this study will give us real-world insights into disease characteristics and various management approaches of patients with MM in the Greater Gulf region.

**International Registered Report Identifier (IRRID):**

DERR1-10.2196/49861

## Introduction

### Overview

Multiple myeloma (MM) is a malignant clonal cancer of plasma cells of the bone marrow that results in an overproduction of huge amounts of light- and heavy-chain monoclonal immunoglobulins [1,2]. In 2020, MM was revealed as the second most predominant hematological malignancy, representing about 10% of hematologic cancers [1], with 176,404 new cases and 117,077 deaths detected worldwide [3]. The hallmark of monoclonal immunoglobulins in MM is the monoclonal protein (M protein), named after its monoclonal properties, detected in patients with MM’s serum and urine [2]. The potential uncontrolled growth of these plasma cells causes destructive bone lesions, kidney injury, anemia, and hypercalcemia [4]. About 15% to 30% of patients have the clinical presentation of hypercalcemia with concomitant renal insufficiency caused by precipitated monoclonal light chains in the collecting tubules [5,6]. However, in asymptomatic patients, the disease is accidentally discovered after detecting anemia or hyperproteinemia [1]. Moreover, around 10% of patients with MM showed a previous history of asthma or chronic obstructive pulmonary disease (COPD). The treatment of MM involves the use of steroids; standard chemotherapy (including cyclophosphamide, melphalan, and bendamustine); proteasome inhibitors such as bortezomib and ixazomib [7]; anti-CD38 monoclonal antibodies (eg, daratumumab and isatuximab), and autologous stem cell transplantation (ASCT) [8].

Guidelines have approved anti-CD38 monoclonal antibodies simultaneously with lenalidomide and dexamethasone for treating relapsed or refractory MM (RRMM) following their remarkable efficacy. Alongside clinical disease complications, the treatment of patients with RRMM relied on 3 key points: the duration of response (DoR), progression-free survival (PFS), and overall survival (OS) reduction with successive lines of therapy [9].

MM shows a socioeconomic burden on the patients as well as their families. Rare data regarding MM in the Middle East have been detected [10]. Nevertheless, the United Arab Emirates (UAE) and Qatar were among the countries with the largest prevalence of MM cases and deaths over the past 30 years across the world [11].

### Rationale

The primary objective of this study is to detect the associated characteristics of patients with RRMM and the treatment landscape in the Greater Gulf region. The secondary objectives are to describe MM disease history; assess the prevalence of lenalidomide-refractoriness among patients with RRMM in the Greater Gulf region; assess the prevalence of renal impairment and asthma or COPD among patients with RRMM in countries of the Greater Gulf region, both at diagnosis and throughout the disease course; describe renal response (overall, per treatment line and regimen for patients with renal impairment); describe the overall treatment outcomes for patients with MM and patients with lenalidomide-refractory (per treatment line and regimen); and reveal the percentage of patients with RRMM who are eligible for ASCT.

## Methods

### Overview

Due to the rarity of MM, this study will be a regional, retrospective study aimed at collecting real-world and epidemiological data from patients with MM’s medical records in countries in the Greater Gulf region. In addition, the study will describe the demographic characteristics and treatment landscape of patients diagnosed with RRMM who have relapsed at least once and maximally 3 times before study entry in the 2 years leading up to data collection. Medical records will be checked for eligibility, and data will be retrieved from all participating countries, including Saudi Arabia, the UAE, Kuwait, Oman, and Qatar.

Participants for whom medical records will be eligible for inclusion, review, and analysis must fulfill the following criteria: male or female patients with RRMM who have relapsed at least once and a maximum of 3 times before study entry in the 2 years leading up to data collection. Patients should be 18 years or older, male or female, diagnosed with RRMM (first, second, and third relapses only, who had 1-3 previous lines of treatment) within a maximum of 2 years before data collection time, and have complete patient medical records from MM diagnosis to date of death or medical abstraction. Patients will be excluded as follows: patients not undergoing treatment for MM or newly diagnosed patients (first-line patients); patients with a history of other malignancies; pregnant patients or those planning for pregnancy; or patients with end-stage renal disease.

This study is based on the secondary use of data; expedited reporting of adverse events or adverse drug reactions is not required. However, the sponsor will report all safety observations made during the conduct of the study in the study report.

All of the data will be obtained from the available records as shown in Textbox 1.

**Study flowchart. All the data will be obtained from the available records. Data will be collected from Saudi Arabia, the United Arab Emirates, Kuwait, Oman, and Qatar.**

**Evaluation or data points in patients’ records**

- *Inclusion and exclusion criteria*
- *Patient characteristics*: nationality, gender, age, and race
- *Multiple myeloma (MM) disease history*: year of diagnosis, diagnostic method used, levels of heavy-chain immunoglobulin, hematological biomarkers, other laboratory findings, and symptoms before diagnosis (if available)
- *Cytogenetic abnormalities*
- *MM-associated morbidities*: renal impairment (estimated glomerular filtration rate values or other kidney function tests) and pulmonary impairment (including asthma and chronic obstructive pulmonary disease)
- *Lines of treatment used*: first-line regimens (at diagnosis), second-line regimens (after first relapse), third-line regimens (after second relapse), and fourth-line regimens (after third relapse)
- *Treatment regimens*: monotherapy or combination therapy
- *Reasons for treatment change or discontinuation*
- *Eligibility for stem cell transplant*
- *Lenalidomide refractoriness status*
- *Treatment outcomes*: dates of progression, relapses, and requiring treatment escalation (due to refractoriness or relapse)
- *Time to progression*
- *Minimum residual disease negativity (if applicable)*

Data sources include—but are not limited to—hospital records (medical, clinic, and pharmacy) and office charts; memoranda; evaluation checklists; laboratory reports, radiology reports, and imaging data (eg, ultrasonography and scans on film or digital media); computer printouts; and any other documentation regarding the patient (including the patient diary).

Based on the study by Lecat et al [12], 71% of patients with RRMM became refractory to lenalidomide after an initial response. Considering an observed percentage of 71% (120.7/170) with an absolute precision of 8% and a 95% CI, a sample size of a minimum of 150 patients was calculated based on the below formula:

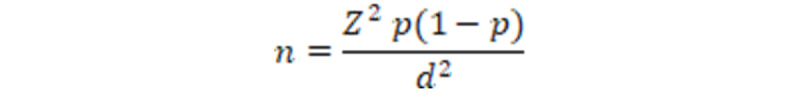



To estimate an observed percentage of 50% (85/170) with an absolute precision of 8% and a 95% CI, the sample size for this retrospective study was estimated to be 170 patients with the assumption that 10% (15/150) of enrolled patients would not be valuable for the primary analyses due to missing values or unfulfilled inclusion and exclusion criteria.

It is planned to review the medical records of the 170 patients from all participating countries, including Saudi Arabia, the UAE, Kuwait, Oman, and Qatar.

The final study will include 15 medical centers distributed as follows: 10 in Saudi Arabia; 3 in the UAE; and 1 medical center each from Qatar, Oman, and Kuwait.

This study is categorized as a Group E study with no applicable individual case safety reporting; however, either aggregate analysis may provide safety conclusions of interest for the product or, even if the analysis is not intended for safety purposes, it may raise a safety signal. Any potential safety signal will be transmitted to the company’s pharmacovigilance department.

All data collected will be analyzed appropriately, and statistical analysis will be carried out by SPSS (version 18 or higher; IBM Corp). The associated characteristics of RRMM will be described using frequency and percentage with a 95% CI. Other variables will be described using the mean (SD) for continuous variables and counts for categorical variables. Patients’ variables will be compared using Mann-Whitney-Wilcoxon tests for continuous variables and chi-square for categorical variables. A probability value of less than 5% (*P*<.05) will be considered significant.

### Primary Analysis

The associated characteristics of RRMM will be described using frequency and percentage with a 95% CI. This analysis will be descriptive and will be conducted on the eligible patients who fulfill the inclusion criteria and who have been selected during the specified study period.

### Secondary Analysis

The disease history for MM will be assessed using frequency and percentage with a 95% CI. This analysis will be descriptive and will be conducted on eligible patients. The incidence of lenalidomide-refractoriness among patients with RRMM in the Greater Gulf region and the prevalence of renal impairment and asthma or COPD among patients with RRMM in countries of the Greater Gulf region will be presented using incidence density rates. Comparison will be done using Mann-Whitney-Wilcoxon tests for continuous parameters and chi-square for categorical parameters. The description of renal response (overall, per treatment line and regimen for patients with renal impairment), the overall treatment outcomes for patients with MM and patients with lenalidomide-refractory (per treatment line and regimen), and the percentage of patients with RRMM who are eligible for ASCT will be described using frequency and percentage. Univariate and multivariate analyses will be conducted on lenalidomide-refractoriness among patients with RRMM in the Greater Gulf region and other variables collected.

This section provides specifications for preparing the final statistical analysis plan, which will be issued before the database lock. Therefore, any differences compared to this statistical section should be identified and documented in the final statistical analysis plan.

### Statistical Analysis

The data will be analyzed using SPSS version 26 software. Categorical variables will be reported as frequency (n) and percentage (%), while quantitative variables will be reported using descriptive measures such as mean, SD, and range. The chi-square test will be used to determine the association of different risk factors with treatment regimens. A value of *P*<.05 to determine the significance level will be used. Multivariate logistic regression analysis will be used to analyze the relationship between the dependent variable and multiple predictors or independent variables. PFS and OS will be used accordingly.

### Ethical Considerations

This study is being conducted in accordance with the principles established by the 18th World Medical Assembly in Helsinki in 1964, along with all subsequent amendments [13]. All patient data will be handled anonymously to ensure patient confidentiality. In accordance with the institutional review board (IRB) approval obtained for this study, there is no requirement for informed consent. The final study will include 15 medical centers, distributed as follows: 10 in Saudi Arabia; 3 in the UAE; and 1 medical center each from Qatar, Oman, and Kuwait. IRB approvals were obtained from the participating countries. In Saudi Arabia, approval was obtained from Mouwasat Hospital under the IRB NCBE- KACST (H-05-D-12) on October 10, 2022; the Directorate of Health Affairs-Aseer Region (REC-15-06-2022) on June 20, 2022; King Faisal Specialist Hospital and Research Center under IRB 2022-47 on June 22, 2022; King Fahad Medical City under 2 hospitals—Al Mana Hospital (King Fahad Medical City approval as an external site, IRB 22-212E in December 2022) and Alkhobar Comprehensive Cancer Center (22-212 IRB 22-212) on July 26, 2022; King Saud University-Medical City under 22/0626/IRB on August 23, 2022; Prince Mohammed bin Nasser Hospital under 22058 on June 28, 2022; and King Abdullah International Medical Research Center-Al Ahsa and King Abdullah International Medical Research Center-Riyadh (IRB: 1519/22) on August 31, 2022. In UAE, approval was obtained from Burjeel Hospital (DOH/CVDC/2022/1473) on August 11, 2022; Cleveland Clinic Abu Dhabi (DOH/CVDC/2022/1622) on November 10, 2022; and Sheikh Shakhbout Medical City, Abu Dhabi, UAE (DOH/CVDC/2022/740) on April 28, 2022. In Oman, approval was obtained from Sultan Qaboos University Hospital (SQU-EC/174/2022 MREC#2801) on July 13, 2022. In Kuwait, approval was obtained from the ethics committee in the Ministry of Health (2022/1988) on May 17, 2022. In Qatar, approval was obtained from the National Center for Cancer Research (MRC-02-22-37) on October 2, 2022.

### Data Analysis

Upon completion of this study, a comprehensive description of the demographic profile of patients with MM, including age, gender, and other relevant data, will be obtained. As well, data on patients undergoing different treatment modalities and regimens, including monotherapy or combination therapy, both overall and for each treatment line, will be collected. Additionally, information on the DoR and time to progression (TTP), both overall and within different treatment lines, will be gathered. This includes assessing the percentage of patients with lenalidomide-refractory in the first, second, and third relapse settings. The study will record the history of MM, including the duration of the disease, laboratory levels of biomarkers, and symptoms before diagnosis. Furthermore, we will investigate the proportion of patients with MM with renal impairment and asthma, COPD, or both at the time of diagnosis and at any point thereafter. These data will be collected for each treatment line and regimen. The changes in estimated glomerular filtration rate and other relevant parameters from baseline to evaluate renal response (whether it remains stable, improves, or worsens) will be analyzed. In addition, information on the proportion of patients prescribed lenalidomide at each treatment line and the treatment regimen for patients with lenalidomide-refractory will be retrieved. TTP and DoR among patients with lenalidomide-refractory in different treatment lines and regimens will be calculated according to the International Myeloma Working Group criteria [2]. For instance, the DoR will be calculated in the subpopulation experiencing treatment response from the date when the response is first met to the date of the first documented progression, whereas the TTP will be calculated as the length of time from the date of diagnosis or the start of treatment for a disease until disease progression. Moreover, the proportion of patients eligible for ASCT at any time after diagnosis will be determined. The definitions of “relapsed” and “refractory” MM have been varied across clinical studies. While some studies have defined “relapsed” as those patients with recurrent malignancies following a remission or patients who were positively affected by rescue therapy yet experienced disease progression during their follow-up with or without maintenance therapy, others have defined patients with “refractory” MM as those who have a failed response (or showed a limited response) to rescue therapy or who progress within 60 days of their last regimen [14,15]. This study will assess the used definitions and their impact on the OS and PFS of the included patients.

### Compliance

RAY-Contract Research Organization (CRO), a third party delegated by Sanofi, is solely responsible for taking all reasonable steps to ensure the proper conduct of the protocol regarding ethics, protocol compliance, and the integrity and validity of the data recorded on the case report form (CRF). The primary responsibility of RAY-CRO is to assist the investigating site in maintaining high standards of ethical, scientific, technical, and regulatory quality throughout the study.

At regular intervals during the study, the site will be contacted, through monitoring visits, letters, or telephone calls, by a representative of the monitoring team to review study progress, investigator and patient compliance with study protocol requirements, and any emergent problems. During these monitoring visits, the following but not exhaustive list of points will be scrutinized by the investigator: IRB approval, documentation and reporting, and quality of data in the CRF.

The main duty of the monitoring team of RAY-CRO will be to assist the investigator and the sponsor in maintaining high ethical, scientific, technical, and regulatory standards throughout the study. Regular monitoring visits, letters, or telephone calls will be made to the site to review study progress, investigator and patient compliance with study protocol requirements, and any emerging issues. During these visits, the investigator will be scrutinized on, but not limited to, the following points: IRB approval, data documentation, and the quality of data in the CRF.

## Results

Patient medical records were reviewed between June 2022 and January 2023 for eligibility and data extraction. A total of 148 patients met the study inclusion criteria, of whom 64.2% (n=95) were male and 35.8% (n=53) were female. The study is currently in its final stages of data analysis, and the final manuscript is expected to be published in 2024.

## Discussion

### Summary

This study is expected to provide reliable data on MM and associated renal and pulmonary impairments in the Greater Gulf region, through the collection of real-world and epidemiological data from the medical records of patients with MM in countries in the Greater Gulf region. In addition, the study will describe the demographic characteristics and treatment landscape of patients diagnosed with RRMM who have relapsed at least once and maximally 3 times before study entry in the 2 years leading up to data collection. All patients who have relapsed at least once and a maximum of 3 times before study entry in the 2 years leading up to data collection will be included. The incidence of lenalidomide-refractoriness among patients with RRMM in the Greater Gulf region, the prevalence of renal impairment and asthma or COPD among patients with RRMM in countries of the Greater Gulf region, renal response, the overall treatment outcomes for patients with MM and patients with lenalidomide-refractory, and the percentage of patients with RRMM who are eligible for ASCT will be presented.

### Expected Outcomes and Comparisons With Previous Work

Although MM has been detected at increasing rates in some countries of the Greater Gulf region [11,16], fewer studies have been conducted regarding low-income countries, including the Middle East and North Africa [17]. Nevertheless, the treatment of MM, largely in relapsed cases, poses major challenges. Although new drugs and other therapeutic interventions enhance the prognosis of MM, the availability of these drugs is limited across the Middle East [18]. Hence, this study will provide an overview of the treatment regimens and associated patient characteristics in Greater Gulf countries. Regardless of updated national guidelines established in the Greater Gulf countries, a deficiency in complete data to guide the decision makers of local patients is detected. Educational gaps arise, and bespoke initiatives are required to help oncologists individualize the treatment and translate the evidence-based endorsements into real-world practice [19,20]. With this study, we hope to elucidate the incidence of lenalidomide-refractoriness among patients with RRMM and the overall treatment outcomes for patients with MM and patients with lenalidomide-refractory (per treatment line and regimen) to guide physicians with the best treatment options and better survival outcomes.

Patients who experienced a ≥25% increase in serum or urine M protein have a biochemical relapse, are considered asymptomatic, and are closely monitored without treatment [21]. In contrast, patients with high-risk disease, including those with negative cytogenetics, suboptimal response to previous treatment, or aggressive disease at diagnosis [22], or those who exhibit a rapid escalation in serum or urine M protein levels, for instance, a doubling time of 2 months or less, should initiate therapy immediately [23]. It is expected that if our patients reveal symptomatic relapse, it should be managed based on the criteria of the International Myeloma Working Group. We hope that this study can provide us with the actual management procedures followed in each country and the consequent responses of those patients.

Regarding the demographic data for MM, age is considered a crucial prognostic factor for patients with MM. Patients aged above 50 years at diagnosis usually exhibit significantly shorter median survival times than younger patients [24]. Nevertheless, recent MM therapy protocols have improved the clinical outcome, particularly in patients younger than 70 years [24,25], while the survival advantage has been recently detected in older patients [26]. This controversial conclusion regarding the improved survival in the older population with MM could be due to the detected variability of the age range included in interventional clinical trials (ie, aged between 60 and 65 years) and the median age of patients with MM at diagnosis (usually around the age of 70 years) in other studies. The median time for diagnosis for these patients usually ranges between 2 and 4 years [23]. According to a study conducted in Jordan, the median age of the included patients was 62 years, the mean OS was 74 months, and the median survival was 38 months [17]. Our anticipated results will provide us with a clear indication of the age range and the influence of age on the prognosis and survival of those patients.

In addition, patients older than the age of 75 years exhibit an increased cumulative incidence of renal insufficiency and cardiovascular disease, from 47.7% at MM diagnosis to 67.8% at the first relapse exhibited. Moreover, in patients with MM aged 75 years or older, the risk of relapse post–second-line therapy increased significantly and alternated by time to the next treatment [11]. Nevertheless, younger patients have exhibited better survival than older populations, particularly those younger than 40 years of age, where they are rarely detected with such comorbid diseases [27]. Additionally, geriatric assessments were recommended to be incorporated into routine clinical practice at the diagnosis of MM and for the exclusion of differential diagnoses in the very elderly. This approach aims to achieve optimum therapy and the adjustment of both dosing and regimens for improved effectiveness and tolerability [28]. As such information is missed among patients with MM in the Greater Gulf countries, this study shall provide us with more details regarding the prognosis of patients aged over 75 years with renal insufficiency, cardiovascular diseases, or both; their management procedure; and the effect of younger age over survival.

For lenalidomide-refractoriness among RRMM, improvement in renal function in patients with RRMM and renal impairment following lenalidomide- or dexamethasone-based treatment has been revealed by many studies [29-31]. Similarly, patients with MM presented with asthma or COPD showed significantly longer time from first- to second-line treatment. Additionally, the OS from first-line therapy was remarkably lower among patients with COPD, with a statistically significant decline in the OS from second-line therapy [32]. As no previous studies in the Middle East and North Africa region have provided the effect of first- or second-line treatment on patients with associated asthma or COPD, this study will assess the OS and PFS in those patients and compare the influence of first- or second-line therapy on survival.

### Limitations

Anticipated limitations of this study include the heterogeneity of management guidelines among the included countries, which may restrict the results obtained from real-world data by reflecting actual practice, influenced by patient compliance and environmental factors rather than solely by the efficacy of the drugs. Furthermore, despite the relative consistency of first-line therapy in the countries of the Gulf Cooperation Council, several different treatment options become available after the first relapse. This study may not be able to comprehensively capture the multiple factors influencing the selection of specific regimens at the first stage of progression. Additionally, the proportion of patients receiving anti-CD38 monoclonal antibody therapy such as daratumumab or isatuximab, which has significantly transformed the MM treatment landscape, remains relatively limited to date.

### Conclusion

This study will provide a more comprehensive understanding of the management of MM in the Greater Gulf region, with a particular focus on the renal and pulmonary complications that may arise. We will delve into the wider significance of this study, addressing potential predisposing factors and their implications for clinical practice and public health in the region. This study seeks to shed light on how the insights gained can inform healthcare strategies and ultimately enhance the quality of care for patients with MM in this specific geographic area.

## Abbreviations

ASCT: autologous stem cell transplantation
COPD: chronic obstructive pulmonary disease
CRF: case report form
DoR: duration of response
IRB: institutional review board
M protein: monoclonal protein
MM: multiple myeloma
OS: overall survival
PFS: progression-free survival
RRMM: relapsed or refractory multiple myeloma
TTP: time to progression
UAE: United Arab Emirates

## Data Availability

Data will be presented in the main manuscript or additional supporting files whenever possible. After completing the study, the data obtained during and/or analyzed during this study will be available upon request through the corresponding author. The authors hereby state that no generative artificial intelligence was used in any portion of the manuscript writing.
